# Hantavirus reservoir *Oligoryzomys longicaudatus *spatial distribution sensitivity to climate change scenarios in Argentine Patagonia

**DOI:** 10.1186/1476-072X-8-44

**Published:** 2009-07-16

**Authors:** Aníbal E Carbajo, Carolina Vera, Paula LM González

**Affiliations:** 1 Unidad de Ecología de Reservorios y Vectores de Parásitos, Depto. Ecología, Genética y Evolución, Facultad de Ciencias Exactas y Naturales, Universidad de Buenos Aires. CONICET, Buenos Aires, Argentina; 2 Centro de Investigaciones del Mar y la Atmósfera (CIMA), Depto. Ciencias de la Atmósfera y los Océanos, Facultad de Ciencias Exactas y Naturales, Universidad de Buenos Aires. CONICET, Buenos Aires, Argentina

## Abstract

**Background:**

*Oligoryzomys longicaudatus *(colilargo) is the rodent responsible for hantavirus pulmonary syndrome (HPS) in Argentine Patagonia. In past decades (1967–1998), trends of precipitation reduction and surface air temperature increase have been observed in western Patagonia. We explore how the potential distribution of the hantavirus reservoir would change under different climate change scenarios based on the observed trends.

**Methods:**

Four scenarios of potential climate change were constructed using temperature and precipitation changes observed in Argentine Patagonia between 1967 and 1998: Scenario 1 assumed no change in precipitation but a temperature trend as observed; scenario 2 assumed no changes in temperature but a precipitation trend as observed; Scenario 3 included changes in both temperature and precipitation trends as observed; Scenario 4 assumed changes in both temperature and precipitation trends as observed but doubled. We used a validated spatial distribution model of *O. longicaudatus *as a function of temperature and precipitation. From the model probability of the rodent presence was calculated for each scenario.

**Results:**

If changes in precipitation follow previous trends, the probability of the colilargo presence would fall in the HPS transmission zone of northern Patagonia. If temperature and precipitation trends remain at current levels for 60 years or double in the future 30 years, the probability of the rodent presence and the associated total area of potential distribution would diminish throughout Patagonia; the areas of potential distribution for colilargos would shift eastwards. These results suggest that future changes in Patagonia climate may lower transmission risk through a reduction in the potential distribution of the rodent reservoir.

**Conclusion:**

According to our model the rates of temperature and precipitation changes observed between 1967 and 1998 may produce significant changes in the rodent distribution in an equivalent period of time only in certain areas. Given that changes maintain for 60 years or double in 30 years, the hantavirus reservoir *Oligoryzomys longicaudatus *may contract its distribution in Argentine Patagonia extensively.

## Background

The colilargo (*Oligoryzomys longicaudatus *Bennet 1832) belongs to a genus of small-sized mice classified in the New World Tribe Oryzomyini (Muridae: Sigmodontinae). It is a widespread rodent primarily found in woods and shrub lands in Chile and southwestern Argentina [1]. This is a sigmodontine rodent of great importance because of its role as a major reservoir for the Andes Sout lineage of hantavirus, which produces hantavirus pulmonary syndrome (HPS) in southern South America [2,3].

HPS is a severe and frequently fatal cardiopulmonary disease [4] caused by a virus of the genus Hantavirus, family Bunyaviridae. The first cases of HPS were reported in the United States in 1993. The etiologic agent, Sin Nombre virus (SNV), was associated with the sigmodontine rodent reservoir, *Peromyscus maniculatus*. Presently, more than 15 variants of hantavirus have been identified in the occidental hemisphere and many countries (Argentina, Bolivia, Chile, Brazil, United States of America, Panama, Paraguay and Uruguay) were affected by HPS with a lethality ranging from 1.8 to 47.2% [5]. In all cases, HPS cases were associated with sigmodontine reservoirs.

Epidemiological analysis and planning preventive measures for zoonotic and vector-borne diseases require a knowledge of the geographic distribution and ecological conditions relevant to the circulation of a pathogen [6]. Study of these factors have been conducted for vector, reservoir and/or disease transmission which helps in the prediction of potential effects of climate change on diseases like malaria, dengue, schistosomiasis, leishmaniasis and chagas [7-10] and their vectors [11].

**Figure 1:**
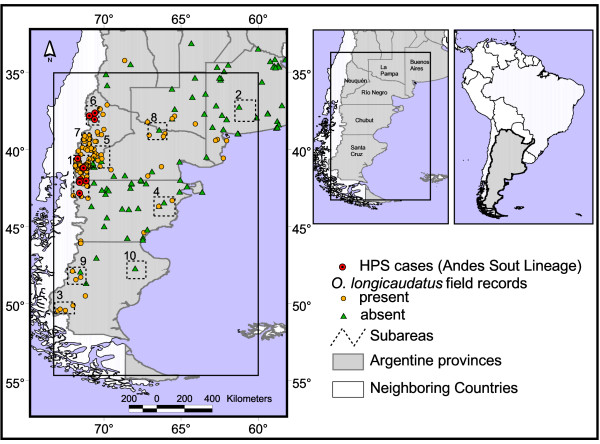
**Study area**. Study area (rectangle), showing Argentine Patagonia and *Oligoryzomys longicaudatus *field records. Dashed lined rectangles and numbers indicate the subareas used to evaluate the changes in *O. longicaudatus *presence probability (see table 1).

Except for a few works in Chile [12,13], the knowledge of colilargo ecology is anecdotal or brief at best. The Patagonian region (Figure 1), has suffered in the last century many environmental challenges that affect the rodent's distribution. Sheep ranching doubled between 1900' and 1950' but has since declined [14]. Fire, considered a natural disturbance that shapes the south Andean forests, has also been influenced by humans since the late eighteen century [15]. Grazing and fire together with temperature and rainfall have a complex relation with multiple feedbacks that influence the establishment and growth of woods, shrubs and grasses [16,17], which in turn affect the colilargo populations. Eruptions in rodent populations (including colilargos) have been associated with bamboo flowering and subsequent seed production as well as with increased rainfall after El Niño [18]. Also population dynamics were associated with the Antarctic Oscillation Index and Southern Oscillation Index in Chile; El Niño events (negative SOI values) had a lagged positive effect on population growth [12]. In the United States not only the abundance of the reservoir but also increased risk of SNV transmission were related to rise in precipitation associated with El Niño [19]. Excepting this last study, most have focused mainly on temporal relationships between climate indexes and population time series. Changes in the spatial distribution, particularly over southern South America, have yet to be explored extensively.

Recently, the spatial distribution of colilargos has been modelled in Argentina as a function of climatic, hydrological and vegetation cover variables [20,21]. The modelling framework used, known as predictive habitat distribution or niche modelling, has rapidly increased in many fields such as biogeography, evolution, ecology, epidemiology, conservation, and invasive-species management [22]. These models associate species and even community occurrences with environmental variables (biotic or abiotic) to predict their potential geographic distribution [23]. The idea is that known occurrences of species across landscapes can be related to raster geographic information system coverages summarizing environmental variation across those landscapes to develop a quantitative picture of the ecologic distribution of the species [24]. Although climate is only one of several factors that influence the distribution of the colilargo, in the Argentine model, mean annual temperature and cumulative precipitation resulted in the best predictors of its spatial distribution [20]. In warmer areas, the presence of colilargos seamed to be associated with lower than normal precipitation values (i.e., north-eastern Patagonia). In colder areas, presence was associated with higher precipitation (i.e., western Andes, close to the Chilean border). The model, being parametric in nature, allows the recalculation of the probability of colilargos presence with different parameters of temperature and precipitation.

**Figure 2:**
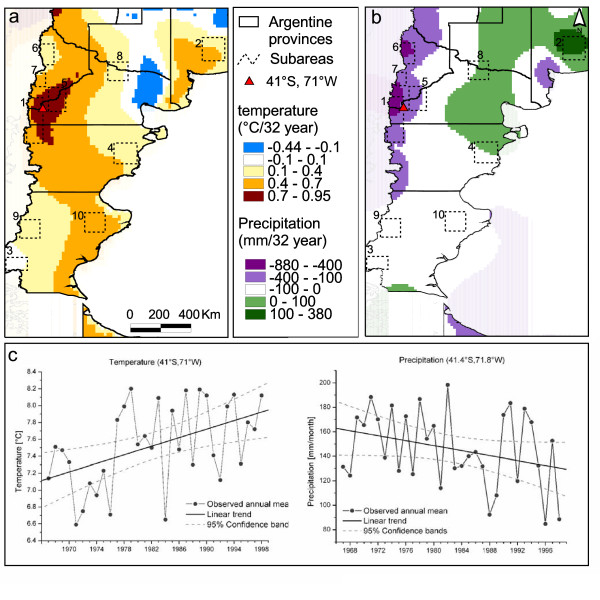
**Temperature and precipitation trends**. Change in mean air temperature (a) and cumulated precipitation (b) observed between 1967 and 1998 in Argentine Patagonia. Time series of temperature (annual average of monthly means) and precipitation (annual average of monthly accumulated rainfall) for the locality indicated in the maps by the red triangle (c).

The Patagonian region, as other regions of the world, has experienced important climate variations during the last century (Figure 2). In particular, significant positive temperature trends in association with negative precipitation trends have been documented along the southern Andes [25,26]. In addition, recent studies estimate climate change projections over South America for the twenty-first century [27] based on information provided by global climate simulations performed under greenhouse gas increment scenarios for the fourth assessment report of the Intergovernmental Panel on Climate Change (IPCC) [28]. There is a general consensus among global climate models that climate changes projected over the southern Andes for the last part of the twenty-first century will be mainly associated with temperature increases and reductions in precipitation during all seasons. Nevertheless, Vera et al. [29], using an ensemble of 21 global climate model simulations, showed that the quantification of such regional potential climate change in the southern part of South America exhibits a large degree of uncertainty that inhibits its use in impact assessment studies. Therefore, the aim of our work is to evaluate the changes in the probability of the colilargo presence that might take place under four different potential climate change scenarios. In particular, we test the hypothesis that the future distribution of the hantavirus reservoir in Argentine Patagonia will be affected if future climate changes of similar magnitude and sign than those already observed in the twentieth century occur. We also examine the sensitivity of the potential distribution of the rodent to the uncertainties associated with those climate change scenarios.

## Methods

The probability of the colilargo presence in a given place (presence probability) was calculated with a validated spatial distribution model which is as a function of temperature and precipitation [20]. To evaluate changes in colilargo distribution its presence probability under current climate was compared to the probability modelled considering four possible future climate change scenarios. The four climate change scenarios were constructed using potential temperature and precipitation changes based on the linear extrapolation into the future of the changes recorded for the period between 1967 and 1998 in the Argentine Patagonia.

### Model

The colilargo distribution model for the Argentinean Patagonia is described in detail elsewhere [20]. Briefly, the distribution model consisted of a generalized linear model [30], using logistic multiple regression. The probability of a site having colilargos (pO) was modelled as an S-shaped curve when LP is a first-order polynomial:

(1)

The linear predictor (PL) was related to the response variable (rv) through the logit link ln(rv/(1-rv)), which linearizes the response and restricts its values to lie between 0 and 1 [31]. The LP was expressed as a linear combination of the environmental explanatory variables (x_i_): LP = a + bx_1 _+ ... + cx_i _+ *e*, with a, b, c being the parameters, and *e *a binomial error term with variance equal to μ (1-μ). The continuous environmental variables tested were mean annual temperature, mean annual cumulative precipitation, mean annual cumulative evapotranspiration, distance to permanent rivers, to any kind of water body, to roads, annual frost-day frequency, elevation above sea level, percent cover by trees, by grass, and by bare soil. The two factor variables tested where the phytogeographic classification of Argentina [32] and a detailed phytogeographic classification of Patagonia [33]. The selection procedure was a manual upward stepwise selection based on a t-test (parameter/standard error with null model degrees of freedom) of each variable with α = 0.01 for retention because of the large number of variables considered. The final model included temperature (tt) and precipitation (pp) as the best predictors and the parameters after bootstrap re-sampling were:

(2)

with txp meaning both variables interaction. The equation was applied to each cell of the study area map with the geographic information system Arcview 3.2 [34]. After transforming the variable with eq (1) the probability of the colilargo being present (presence probability) was obtained. Values lower than 0.5 indicated absence and values above or equal to 0.5 indicated presence. The model explained 59% of the total variance in presence and the external validation resulted in an 86% correct classification of the colilargo records [20].

### Climate change scenarios

Considering that the best current distribution model was a function of temperature and precipitation, the climate change scenarios were built considering these two variables. Furthermore, as it was mentioned before, there is currently a lack of reliable quantification of future climate change at regional levels [28]. In that sense, considering that IPCC reports and publications like [27] estimate that climate changes projected in Patagonia for the twenty-first century will be of the same sign than those observed in the second portion of the twentieth century, climate change scenarios were built considering the climate trends observed in the region during the last portion of the twentieth century.

Monthly mean surface air temperature from the Climatic Research Unit dataset [35] along with a gridded dataset of monthly rainfall means derived from meteorological stations were used to create the model. The linear trends of both variables were computed over the period 1967–1998 using a least squares technique. That period is long enough to estimate trends besides the climate variations associated with the natural climate variability. Those trends were mainly characterized by a generalized warming over the whole region with peaks along the Andes region (Figure 2a), and a significant decrease of precipitation along the Río Negro and Neuquén mountains (Figure 2b).

As mentioned in the Introduction, current global climate models project for the twenty-first century under a greenhouse gas increment scenario trends in both temperature and precipitation of the same sign as those observed during the last part of the twentieth century. Nevertheless, those climate models do not provide a realistic quantification of such change, due to the uncertainties inherent in such projections [29]. Therefore, climate change scenarios for the region under study were estimated assuming that climatic trends observed over the period 1967–1998 would continue into the future. Based on the assumption of continued climate change, four different climate change scenarios were constructed: Scenario 1 assumed no change in precipitation but a future temperature change equal to that observed between 1967 and 1998; Scenario 2 assumed no change in temperature but a future precipitation change equal to that observed between 1967 and 1998; Scenario 3 assumed changes in both variables equal to those observed between 1967 and 1998; Scenario 4 assumed changes in both variables but doubled the values observed between 1967 and 1998.

### Procedure

Equations (1) and (2) of the distribution model were calculated cell by cell based on each scenario over the whole of Patagonia (square cells of approximately 19 km side). This created four maps of colilargo presence probability. To obtain a clearer view of presence probability change, the original map (obtained from 1967–1998 climate conditions) was subtracted from each scenario map (scenario model pO minus original model pO). This substraction of probabilities rendered 4 maps showing the "change in presence probability," positive values indicated an increase in presence probability according to the scenario and negative values a decrease.

To test the hypothesis of no change in presence probability due to the postulated climate change in the whole Patagonia, 230 random sites were sampled from the original model and from the four scenarios of climate change. A pair wise comparison between the original model and each scenario was used to assess significant differences in presence probability (Wilcoxon Rank Sum test). To determine if these changes could be spatially heterogeneous, ten homogeneous sub-areas were arbitrarily selected to evaluate local changes (Figure 1). Changes were analysed with a test for differences of proportions [36]. The sub-areas were chosen in order to contain hantavirus transmission zones (1 & 6), zones without transmission but with the rodent widely present (3, 5, 7, 9), zones without transmission but with the rodent sparsely present (4, 8) and zones without transmission or rodent presence (2 & 10). The data about transmission corresponds to records between 1989 and 2002. After that date only the hospital of treatment was available, but potential place of infection and place of residence of the patient were not. The location of 59 cases out of 76 could be geolocated according to the potential place of infection. Sites separated by less than 5 km were clustered together resulting in 12 separate points. Finally, total areas of potential presence were compared between the original model and the significant scenarios. It was assumed that pixels with presence probability equal or higher than 0.5 indicated potential presence of the rodent, and those below 0.5 indicated potential absence. A contingency table was built with the number of pixels potentially present and potentially absent in the original model versus the scenario model. The differences were assessed with a χ^2 ^test.

## Results

**Figure 3:**
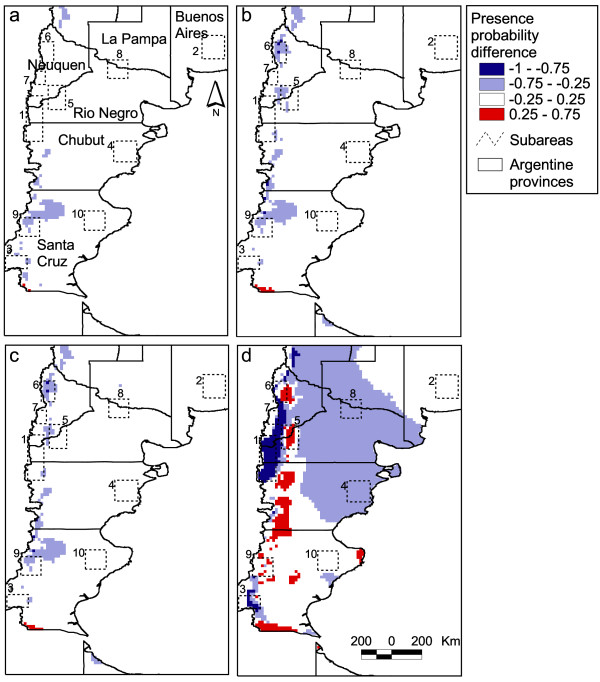
***Oligoryzomys longicaudatus *presence probability change**. *Oligoryzomys longicaudatus *presence probability change according to temperature and precipitation change scenarios. The maps show the difference between scenarios probability and the actual presence probability. a- Scenario 1, temperature change alone; b- Scenario 2, precipitation change alone; c- Scenario 3, temperature and precipitation change; d- Scenario 4, temperature and precipitation change twofold. Positive values indicate an increase in presence probability.

The colilargo presence probabilities were different among the four climate change scenarios, as shown in Figure 3. The presence probability based on Scenario 1 decreased in western Chubut and Santa Cruz provinces. Scenarios 2 and 3 also showed a decrease in those same areas as well as in western Neuquén. A rise in probability was observed in Scenarios 2 and 3 in a small patch in south-western Santa Cruz. On the other hand, Scenario 4 showed the greatest changes in presence probability. These changes occurred in the Andes range and in a fringe crossing the northern Patagonia from northwest to centre east. Rodent presence probability increased to 0.75 in patches aligned north-south from Neuquén to southern Santa Cruz. The similarities observed between scenarios 2 and 3 suggest that changes in precipitation may have a stronger effect on presence probability than temperature alone (Scenario 1).

According to the randomly sampled points only scenario 4 presented significant changes in presence probability (Z = -10.773, p < 0.0000). Thus the hypothesis of no change in presence probability is rejected for Scenario 4, which assumes a doubling of values in the trend observed between 1967 and 1998. The analysis for the sub-areas showed only significant reductions in presence probability (table 1). Scenario 4 showed most of the changes, with 7 out of 10 areas exhibiting significant probability decreases. Under this scenario the transmission area in Río Negro experienced a decrease in its colilargo presence probability by 87% (actual mean 0.997; Scenario 4 mean 0.129). Curiously, the only subset area that showed significant changes in another scenario was the transmission zone in Neuquén, where a fall of nearly 50% in colilargo presence probability was observed for scenarios 2 and 3 (actual mean 0.812; Scenario 2 mean 0.381 and Scenario 3 mean 0.412).

**Table 1:** Presence probability comparison. Differences in *Oligoryzomys longicaudatus *presence probability between four scenarios of climate change and actual conditions.

Original model presence probability	Sub area	Scenario			
		1	2	3	4
1.00	1^1^	0.00	-0.02	-0.03	-0.87**
0.08	2^2^	-0.01	-0.04	-0.06	-0.08*
0.81	3^3^	-0.03	0.00	0.00	-0.33**
0.50	4^4^	0.04	0.02	0.04	-0.50**
0.54	5^3^	0.00	-0.14	-0.07	0.03
0.81	6^1^	-0.05	-0.43**	-0.40**	-0.09
1.00	7^3^	0.00	0.00	0.00	-0.17*
0.69	8^4^	0.04	-0.07	-0.06	-0.69**
0.63	9^3^	-0.16	-0.16	-0.16	0.06
0.33	10^2^	0.05	0.01	0.05	-0.15*

^1^Areas where HPS cases have occurred

^2^No HPS cases, no rodent

^3^No HPS cases, rodent widespread

^4^No HPS cases, rodent scarce.

Positive values indicate an increase in presence probability. The presence probability according to the original model is shown in the first column (probabilities below 0.5 coincide with rodent actual absence). The subareas numbers indicate the corresponding areas in Figure 1. Scenario 1, temperature change alone (see text for details); Scenario 2 precipitation change; Scenario 3 temperature and precipitation change; Scenario 4 temperature and precipitation change twofold. Significant differences are indicated by * p < 0.05 and ** p < 0.01.

According to Scenario 4, the total surface of potential presence and potential absence of the rodent changed significantly (χ^2 ^= 247.02, df = 1, p < 0.0001). 418,000 km^2 ^would change from potential presence to absence and 82,000 km^2 ^from potential absence to presence. This would imply a net reduction in potential presence surface of 336,000 km^2^. On the other hand, 120,000 km^2 ^of potential presence and 1,106,000 of potential absence will not change their state (72% of the surface in total).

## Discussion

In this work, future potential distribution changes of the geographical distribution of a hantavirus reservoir, colilargo, were built assuming different climate change scenarios. These scenarios were estimated by extrapolating the climate trends observed in the Argentine Patagonia over the last part of the twentieth century. We used scenarios with spatially heterogeneous changes instead of a constant temperature or precipitation change for the whole study area. This proved to be important because a single value for a whole area might hide important local clusters of high presence probability. For example, using the mean precipitation change for Patagonia (20 mm) would have concealed rises of 380 mm and falls of nearly 900 mm in certain areas. These local changes were important, because they coincided with known areas of high transmission.

The starting point of this work was based on historical records of the species recorded during a long time span; we used the best information available about the rodent and the HPS cases. The temperature and precipitation data used therein did not exactly match the records compilation time. The model also assumed a static distribution of the species (i.e., in equilibrium with the environment), an assumption which is usually permitted for modelling purposes in spite of the large spatial extent of the study [23]. It would be desirable to increase the temporal resolution of the rodent monitoring [12,13,37,38] and to incorporate more variables in a study of transmission risk, including the relation between climate, fire, vegetation growth and seedling, rodent population dynamics [13] and hantavirus cases directly [39]. But this is a difficult task to achieve in widespread areas and into the future, as it involves sequential relations (i.e., rain, flowering, seed release, population explosions) combined with spatially separated events (fires, cattle ranching, mast year) and many interactions. For example, in South America bamboo blooming events happen regularly, they occur every 12–14 years in a region but only repeat every 60 years in a certain spot [18]. They not only influence rodent outbreaks but also affect the establishment of woods through favouring of fires and herbivory of the rodents on the seedlings [40]. Also in southern Chile wood exploitation and plantations have produced landscape fragmentation which favours bamboo growth [41]. Until spatio-temporal models together with extensive sampling are put together to include these variables and interactions we believe a summarizing model like the one presented herein is a step forward.

Colilargos are known to inhabit subantarctic forests, where woodlands are abundant, but they also occupy the steppe along scrublands adjacent to streams and roads [42].

Precipitation has a strong influence on the distribution of these woodlands, which might explain the observed subtle dominance of precipitation over temperature in influencing the distribution of the rodent. In Scenario 4 the higher presence probability areas for colilargos are predicted to shift eastwards, while the areas in the north eastern Patagonian steppe would become potential absence. This result can be explained by the combined changes of temperature and precipitation. In the western Andes temperature would rise and precipitation fall, probably diminishing the suitability of the environment for the rodent. However, to the east of these areas, the effects would result in the opposite (less augmentation of temperature and a rise in precipitation).

The relation between the colilargo distribution and HPS transmission is not straightforward. Colilargo populations experience dramatic outbreaks associated to bamboo blooming and mast seeding in southern Chile and Argentina; thus bamboo flowering may be used as a signal for forecasting colilargo outbreaks to predict transmission risk [12]. But although in Chile these outbreaks were associated with an increased risk of HPS [3], in Argentina no cases were detected during an outbreak of colilargos in 1997 [37]. In this same study there was no apparent correlation of rodent antibody prevalence with population density, or of rodent population density or antibody prevalence with numbers of human cases. Regarding human exposition risk, in Europe milder weather has been mentioned as a possible cause of more outdoor activities (recreational and occupational) and thus higher human exposure to infectious rodent excretions [43]. Provided this temporal relation also held spatially, one could expect higher exposition in warmer areas.

In northern Argentina rural occupation was associated with higher hantavirus antibody prevalence, specially agricultural activities [44]. But in the four corners U.S.A. most exposures occurred in or around the home [45]. If we only consider studies about the Andes Sout lineage, in Chile the higher number of rodents positive for the virus were captured also in the domiciles and their surroundings [41]. In southern Argentina seropositive colilargos were found in peridomestic rural environments [46] and a higher risk was anticipated in rural areas due to the lower trap success in the periurban areas [37]. Also the subantarctic forest was the only ecosystem that harboured seropositive rodents in sylvan areas [37,47]. Therefore the probability of humans contracting HPS may be greater in forest habitats, which is actually where most HPS cases have occurred. The distribution of reservoir species indicate the maximum potential extent of HPS [48] and in Argentine Patagonia HPS transmission zones matched the areas with the highest probability of colilargo presence [20]. These findings suggest that presence probability of colilargos may indicate an approximate risk of transmission.

Our results suggest that if climate trends in the twenty-first century continue at the same rates as observed during the last portion of the twentieth century, significant changes in colilargo presence probability would be expected in 30 years from now in certain areas, and more extensively in 60 years (28% of the study area). The condition found with the Scenario 4 may be seen as either a steady maintenance of trends for the future 60 years or their doubling for the next 30 years. These results provide an estimate of the sensibility of the model, and suggest possible time periods that may be needed to observe changes in the rodent distribution under the assumptions of this work.

Also, the decline in presence probability of colilargos in the areas with HPS transmission and the net reduction in total area of potential presence suggest that future changes in Patagonia climate may lower transmission risk. In IPCC [28], the possibility of the incidence of some diseases decreasing due to climate change is mentioned only marginally. Most of the attention is centred on diseases that would increase their risk of exposure. Here we present a situation, in which, future potential climate change might reduce the extent of the distribution of a hantavirus reservoir and maybe the risk of its transmission.

## Conclusion

Provided that temperature and precipitation follow the trends observed between 1963 and 1998 in Argentine Patagonia, the presence probability of *O. longicaudatus *would drop in some areas. Given that changes maintain for 60 years or double in 30 years, the hantavirus reservoir would contract its distribution, and although favourable areas might shift position eastwards, the net total area of potential presence would also fall.

## Competing interests

The authors declare that they have no competing interests.

## Authors' contributions

AEC and CV designed the study. CV and PLG prepared and processed climatic data and maps. AEC made the modeling. All authors interpreted results, read and approved the final manuscript.
